# Conformational antigenic heterogeneity as a cause of the persistent fraction in HIV-1 neutralization

**DOI:** 10.21203/rs.3.rs-2613503/v1

**Published:** 2023-02-24

**Authors:** Philippe Colin, Rajesh P. Ringe, Anila Yasmeen, Gabriel Ozorowski, Thomas J. Ketas, Wen-Hsin Lee, Andrew B. Ward, John P. Moore, P.J. Klasse

**Affiliations:** Cornell University; Cornell University; Cornell University; The Scripps Research Institute; Cornell University; The Scripps Research Institute; The Scripps Research Institute; Cornell University; Cornell University

**Keywords:** HIV-1 neutralization, broadly active neutralizing antibodies (bNAbs), persistent fraction, efficacy, antigenic heterogeneity, stoichiometry, binding kinetics

## Abstract

**Background:**

Neutralizing antibodies (NAbs) protect against HIV-1 acquisition in animal models and show promise in treatment of infection. They act by binding to the viral envelope glycoprotein (Env), thereby blocking its receptor interactions and fusogenic function. The potency of neutralization is largely determined by affinity. Less well explained is the persistent fraction, the plateau of remaining infectivity at the highest antibody concentrations.

**Results:**

We observed different persistent fractions for NAb neutralization of pseudovirus derived from two Tier-2 isolates of HIV-1, BG505 (Clade A) and B41 (Clade B): it was pronounced for B41 but not BG505 neutralization by NAb PGT151, directed to the interface between the outer and transmembrane subunits of Env, but negligible for either virus by NAb PGT145 to an apical epitope. Autologous neutralization by poly- and monoclonal NAbs from rabbits immunized with soluble native-like B41 trimer also left substantial persistent fractions. These NAbs largely target a cluster of epitopes in a hole in the dense glycan shield of Env around residue 289. We partially depleted B41-virion populations by incubating them with PGT145- or PGT151-conjugated beads. Each depletion reduced the sensitivity to the depleting NAb and enhanced it to the other. Autologous neutralization by the rabbit NAbs was reduced for PGT145-depleted and enhanced for PGT151-depleted B41 pseudovirus. Those changes in sensitivity encompassed both potency and the persistent fraction. We then compared soluble native-like BG505 and B41 Env trimers affinity-purified by one of three NAbs: 2G12, PGT145, or PGT151. Surface plasmon resonance showed differences among the fractions in antigenicity, including kinetics and stoichiometry, congruently with the differential neutralization. The large persistent fraction after PGT151 neutralization of B41 was attributable to low stoichiometry, which we explained structurally by the conformational plasticity of B41 Env.

**Conclusion:**

Distinct antigenic forms even of clonal HIV-1 Env, detectable among soluble native-like trimer molecules, are distributed over virions and may profoundly mold neutralization of certain isolates by certain NAbs. Affinity purifications with some antibodies may yield immunogens that preferentially expose epitopes for broadly active NAbs, while shielding less cross-reactive ones. NAbs reactive with multiple conformers will together reduce the persistent fraction after passive and active immunization.

## Background

Neutralizing antibodies (NAbs), whether induced by infection or vaccination, are the best correlate of protection against viral infections in general [1, 2]. Neutralization is defined as interrupting the viral replicative cycle before the first virally encoded transcriptional event by the binding of the neutralizing agent to the virion surface [3]. The mechanism of neutralization of enveloped viruses, as far as is known, is always a direct or indirect block of any step in the entry process, such as receptor interaction and fusion of the envelope with a cellular membrane [3]. To mediate that block, a certain occupancy of NAbs on the virions is necessary and sufficient [3, 4].

The elicitation of broadly active NAbs (bNAbs), capable of neutralizing most circulating variants of HIV-1, remains a central albeit elusive goal of vaccine development [5–12]. The difficulties arise from a number of defenses against neutralizing responses that the HIV-1 envelope glycoprotein (Env), the sole target for NAbs, has developed: extreme sequence variability in surface-exposed regions of the protein, poor reactivity with germline B-cell receptors, conformational and oligomeric masking of functionally important sites, and a malleable glycan shield [9, 10, 13, 14]. Holes in the glycan shield on the Env immunogen due to absence of glycosylation sites or the underoccupancy on actual sites tend to be targeted by narrow autologous responses [15–21].

Soluble Env trimers of the SOSIP.664 design derived from the BG505 (Clade A) and B41 (Clade B) HIV-1 isolates have been shown by crystallography and cryo-electron microscopy (EM) at high resolution to adopt near-native structures and to expose bNAb epitopes preferentially [22–30]. We used both of these trimers to investigate binding parameters that might explain differences in neutralization plateaus for the corresponding two viruses.

Studies of NAbs uniformly measure their potency, *i.e*., the concentration or dilution that gives a certain reduction of the viral infectivity; more neglected is the efficacy of neutralization, *i.e*., the maximum inhibition achieved at the highest NAb concentrations, or its converse, dubbed the persistent fraction (PF) [3, 31–34]. This plateau of the neutralization curve is sometimes obvious and well below 100%; in other cases log-log plots of relative infectivity as a function of NAb concentration can reveal substantial PFs that remain hidden in traditional plots with % neutralization on the y axis [3].

We analyzed the neutralization potency and efficacy of three bNAbs, 2G12 (V3-oligomannose epitope [35]), PGT145 (trimer-specific apical epitope [36, 37]), and PGT151 (gp120-gp41-interface epitope [38, 39]) against two Env-pseudotyped viruses (PVs): BG505 [24, 40] and B41 [25]. We compared these quantitative neutralization properties with the kinetics, affinities, and stoichiometries of binding to SOSIP trimers derived from the same isolates. And we detected antigenic heterogeneity by affinity fractionation of both PV virions and SOSIP trimers, thereby explaining contributions to the PF that apply even to clonal, *i.e*., genetically homogeneous virus (cf. [41]).

Progress towards eliciting bNAbs by active vaccination was recently made through such strategies as germline targeting, immunogen presentation on virus-like particles, sequential immunization, and mRNA delivery [15, 16, 19, 42]. Once bNAbs can be induced, their efficacy can arguably be as important as their potency in preventing transmission [3, 43]. Furthermore, bNAb therapy is showing great promise [8, 13, 14, 44, 45]. In line with our findings, combinations of bNAbs in passive immunization and aiming for multiple specificities in active immunization are rational strategies for counteracting antigenic heterogeneity and thereby reducing the PF, which could otherwise undermine therapeutic and preventive success [3, 4, 6, 46].

## Results

### Comparative neutralization potency and efficacy

**Figure 1:**
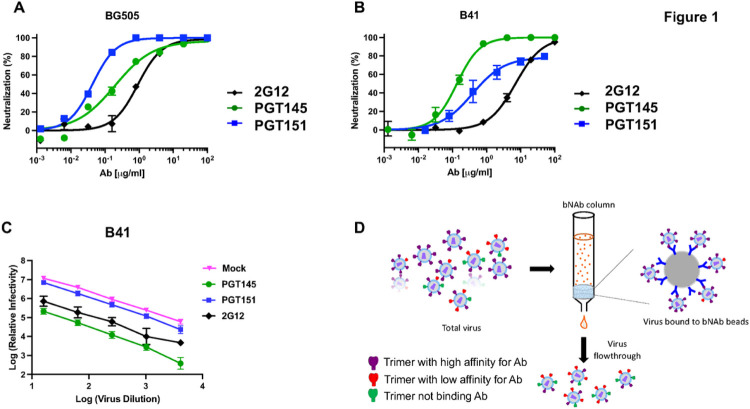
Neutralization efficacy of BG505 and B41 pseudovirus. The extent of neutralization (%) of PV in a TZM-bl assay is plotted as a function of NAb concentration (μg/ml); means+/−s.e.m. of 2–4 replicate titrations are shown. Neutralization by the three bNAbs 2G12 (N332 glycan), PGT145 (V2-apex), and PGT151 (gp41-gp120 interface) of BG505 PV (**A**) and B41 (**B**) gave different potencies and efficacies. **C**. The log_10_ of the relative remaining infectivity after neutralization by bNAb at a fixed concentration (100 μg/ml) is plotted as a function of the log_10_ of varied viral inoculum dilution. **D**. The schematic shows the procedure for partial depletion of a PV preparation by the absorption of virions binding most avidly to bNAbs on beads.

A comparison of neutralization by the three bNAbs 2G12, PGT145, and PGT151 showed differential potencies and efficacies against BG505 and B41 PV. All three bNAbs left negligible PFs of BG505 PV. Similar efficacies notwithstanding, the potencies differed (IC_50_ was 0.91 μg/ml for 2G12, 0.20 μg/ml for PGT145, and 0.043 μg/ml for PGT151). The potency ranking from highest to lowest for BG505 was thus: PGT151, PGT145, and 2G12 (Fig. 1A). In contrast, B41 PV was neutralized to close to 100% by 2G12 and PGT145, but PGT151 left a PF of 21%. The potencies differed also against B41 (IC_50_ was 7.0 μg/ml for 2G12, 0.14 μg/ml for PGT145, and 0.39 μg/ml for PGT151), thus ranking downwards in a different order: PGT145, PGT151, and 2G12 (Fig. 1B).

An excessive amount of the viral antigen that is the target of NAbs would be a potential cause of incomplete neutralization. Through specific adsorption, the concentration of free NAb would then decline Significantly so that it approaches or falls below the dissociation constant, K_D_. Then the ensuing occupancies would not be predicted by the ratio of the added NAb concentration over the K_D_. If the virus inoculum is sufficiently small, however, the decline in concentration of free NAb is negligible and the proportion of virus that is neutralized remains approximately constant over a range of inoculum doses. Figure 1C shows the titration of virus against a high, constant concentration of bNAb (50 μg/ml). The drop in log_10_ infectivity signal was approximately constant for the three bNAbs over a wide range of viral input. The neutralization with varied bNAb concentration and varied viral input together also illustrate the partial independence of potency and efficacy: PGT151 was more potent than 2G12 before reaching the PF, but 2G12 was the more effective of the two (Fig. 1B and C). Insufficient bNAb amount relative to virus is thus ruled out as a contributor to the PF: the relative neutralization was constant over a wide range of viral input and thus adheres to the percentage law of neutralization [47].

### Antigenic heterogeneity of Env spikes on PV

To dissect antigenic heterogeneity in the PV population, we conjugated bNAbs to Sepharose beads to deplete inocula of virions most reactive with the respective bNAb (Fig. 1C). The depletion is designed to be partial; the number and density of Env spikes with at least some antigenicity will determine the avidity of virion capture (Fig. 1C). Meaningful depletions were possible only for B41 PV, which showed the substantial PF; depletion of BG505 PV left negligible and insufficient infectivity for a mechanistic dissection.

**Figure 2:**
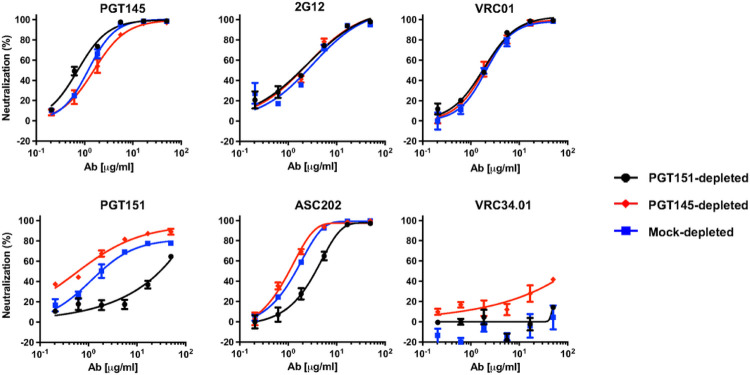
Neutralization of bNAb-depleted B41 PV by bNAbs. The extent of neutralization (%) is plotted as a function of bNAb concentration (μg/ml). PV was first incubated with Sepharose beads covalently conjugated with bNAbs PGT145 or PGT151 or mock-conjugated (see color-coded legend). The unbound virions were then tested for neutralization by the NAbs indicated in each diagram in a TZM-bl neutralization assay. The extent of neutralization (%) is plotted as a function of bNAb concentration (μg/ml), so that potencies rise as curves are shifted from right to left.

Henceforth, we focus on B41 neutralization potency and efficacy, in particular seeking explanations in antigenic heterogeneity of its large PF with PGT151. Depletion of the B41 PV by PGT145- and PGT151-conjugated beads gave differentially neutralized fractions (Fig. 2). Neutralization by PGT145 was consistently but only moderately more potent for PGT151- than PGT145-depleted PV, mock-depleted PV falling in between; the neutralization efficacies were close to 100% after the three depletions (Fig. 2). Neither neutralization by 2G12 nor VRC01, directed to a CD4-binding-site (CD4bs) epitope, was affected by the depletions. In contrast, PGT151 neutralization was 2 orders of magnitude more potent against PGT145- than PGT151-depleted PV, the potency for mock-depleted PV falling between, somewhat closer to the former than the latter. The corresponding PF was reduced for PGT145- compared with mock-depleted PV, whereas the PGT151 neutralization of PGT151-depleted PV was so diminished that no PF plateau could accurately be extrapolated. ASC202, directed to an interface epitope overlapping that for PGT151, neutralized with potencies ranking as for PGT151 against the three depleted PV fractions but with smaller shifts and without yielding detectable PFs for any depletion. Finally, N123-VRC34.01 (hereafter VRC34.01), also specific for an interface epitope [48], weakly and partially neutralized the PGT145-depleted PV (40% at 50 μg/ml) but had no effect against the other two fractions.

**Figure 3:**
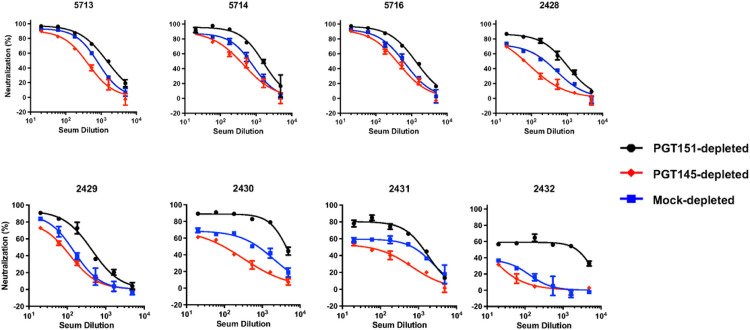
Neutralization of bNAb-depleted B41 PV by sera from immunized rabbits. The extent of neutralization (%) of PV depleted as in Figure 1C and 2 is plotted as a function of serum dilution factor. Thus, in contrast to diagrams for monoclonal Abs, the potency rises as curves are shifted from left to right.

Eight sera from B41 SOSIP.664-immunized rabbits [17, 49] yielded a range of PFs against mock-depleted PV: 5–60% (Fig. 3). Neutralization potency and efficacy were consistently lower against the PGT145-depleted than the PGT151-depleted PV, the curves for the mock-depleted PV falling in between. Just as the sizes of the PFs varied greatly among the sera, however, so did the differences in PFs with each serum for the three depletions.

**Figure 4:**
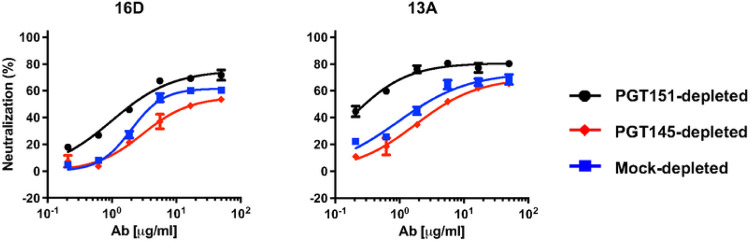
Neutralization of bNAb-depleted B41 PV by mNAbs from immunized rabbits. The extent of neutralization (%) of PV depleted as in Figure 1C, 2, and 3 is plotted as a function of autologous mNAb concentration (μg/ml). In contrast to the plots for the sera in Figure 3, the potency rises as curves are shifted from right to left.

Two monoclonal NAbs (mNAbs), 13A and 16D, were isolated from two of these B41-immunized rabbits (5713 and 5716 [50, 51]). Like the sera, they neutralize the autologous Tier-2 PV well, and the neutralization of a mutant with a knock-in N289-glycan is markedly reduced [50, 51]. Congruently with the results for the sera, neutralization potency and efficacy were lower against the PGT145-depleted than the PGT151-depleted PV, whereas curves for the mock-depleted PV fell in between; the corresponding PF differences were clear with both mNAbs (Fig. 4). Although it is not feasible to elute infectious PV from the bNAbs on the beads, the depletion results suggest that the more PGT151-reactive Env on the PV virions, which preferentially tethers them to the beads, exposes this off-target glycan-hole epitope less well than does the more PGT145-reactive Env.

### Differential purification and antigenicity of soluble native-like Env SOSIP.664 trimers

**Figure 5:**
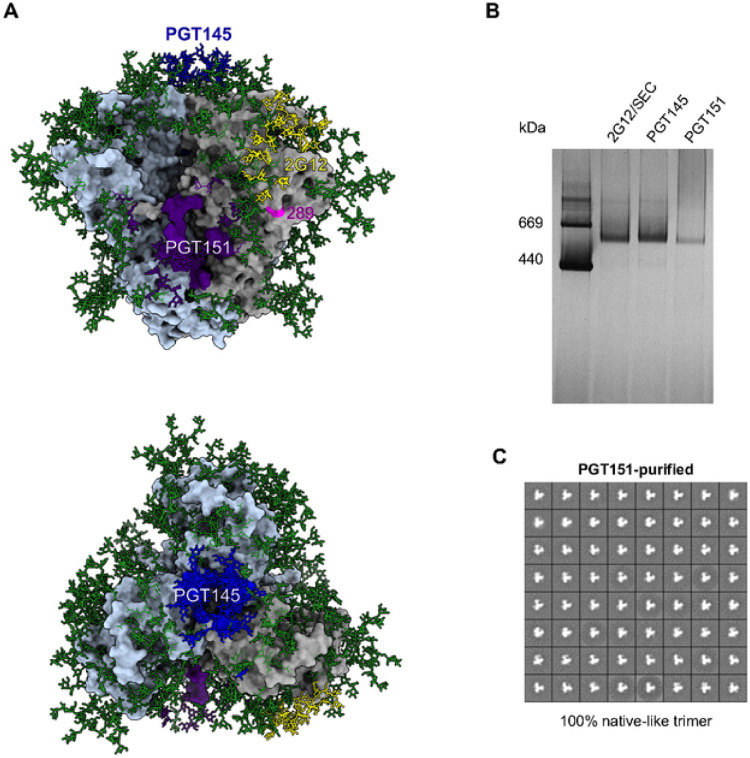
B41 SOSIP.664 trimer. **A.** A surface-rendered model of the B41 SOSIP.664 trimer (PDB 6MCO [52]) oriented with the apex up (top model) or viewed from above (bottom model). The peptidic surface is gray on one protomer and light blue on the other two. Man_9_ glycans were added to the published model [52] and are depicted as sticks and colored green unless they are directly involved in the epitopes of the three bNAbs used for affinity purification, in which case they are colored as the rest of the epitope. Contacts with PGT145 are colored blue, with 2G12 yellow, and with PGT151 purple. N289, which is not part of a PNGS in B41 SOSIP.664, is colored magenta. **B**. Purified Env proteins were analyzed by electrophoresis in 4–12% Bis-Tris BN-PAGE gels with Coomassie-blue staining. 2μg protein per well was loaded from each purification. C. NS-EM analyses of unliganded B41 SOSIP.664 trimers purified by PGT151-affinity chromatography. The propeller-like, triangular particles show 100% native-like trimers.

The 2G12, PGT145, and PGT151 epitopes, as well as the N289 residue, which is part of a glycan-hole epitope on wild-type B41 Env, are shown in their oligomeric-structural contexts in Fig. 5A. The model is based on the crystal structure of the B41 SOSIP.664 trimer, with the addition of Man_9_ oligomannose glycans to all potential N-linked glycosylation sites (PNGS) [50, 52]. Figure 5A illustrates how the glycan-knock-in mutation at N289 fills a defect in the glycan shield, thereby blocking a prominent epitope for autologous NAbs [49]. That soluble trimers with structures similar to the native one shown in Fig. 5A can be obtained by 2G12- and PGT145-affinity purification has been shown multiple times for BG505 and B41 SOSIP.664 trimers by negative-stain electron microscopy (NS-EM) [17, 22–25, 30, 53]. As found here, the PGT151-purified B41 SOSIP.664 trimer also appeared trimeric by BN-PAGE (Fig. 5B). NS-EM 2D-class averages revealed intact trimer molecules with overall movement of the 3 protomeric lobes relative to the center, suggesting substantial conformational “breathing” within the basic native structure, as is typical for B41 trimers (Fig. 5C) [25]. Particles lacking a central triangular mass, a defect that characterizes non-native structures, such as those of uncleaved Env [54], were not observed. We have thus demonstrated that PGT151-affinity purification also yields native-like B41 SOSIP.664 trimer molecules, which enabled us to perform bNAb-binding analyses with differentially purified trimers and explore the antigenic heterogeneity further.

In pursuit of explanations of the large PF specifically with PGT151 against B41 PV, we first used ELISA to explore the binding of bNAbs to B41 SOSIP.664 trimer purified by 2G12-, PGT145-, or PGT151-affinity chromatography and thereafter by SEC (**SI** Fig. 1). 2G12 bound similarly to the three trimer preparations. PGT145 bound strongly to PGT145- and 2G12-purified trimers but considerably less well to PGT151-purified trimer. Conversely, PGT151 bound strongly only to the trimer purified with PGT151 itself and weakly to 2G12- and PGT145-purified trimer. The differential binding of VRC34.01, which like PGT151 is directed to an interface epitope [48], was similar to that of PGT151. The two autologous rabbit mNAbs resembled each other in binding profile: strong binding to PGT145- and 2G12-purified, and substantially weaker to PGT151-purified trimer. These results suggest that PGT151 has a high affinity for a subpopulation of the antigenically heterogeneous trimer molecules, which exposes the 289-glycan-hole epitope less well than the most PGT145-reactive subpopulation.

**Figure 6:**
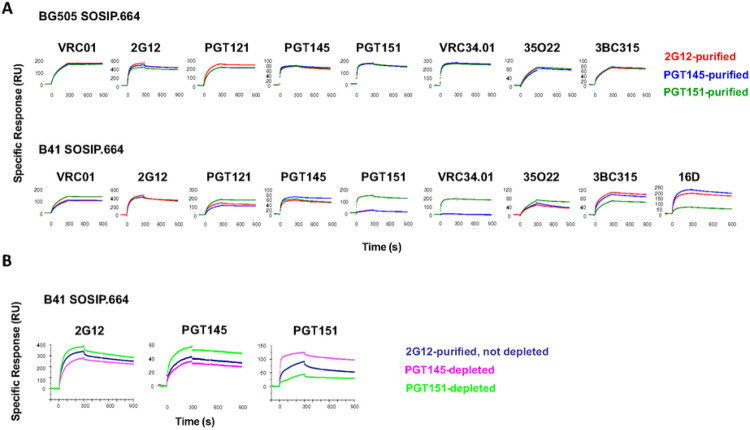
SPR analysis of bNAb binding to bNAb-purified and -depleted BG505 and B41 SOSIP.664. Sensorgrams for individual bNAbs are shown. **A**. BG505 (top) and B41 (bottom) SOSIP.664 trimers were affinity-purified the bNAbs 2G12, PGT151, or PGT145 in a first step and thereafter by SEC. To the right in the B41 row is the sensorgam for the autologously neutralizing rabbit mNAb 16D. Each sensorgram shows the response after background subtraction on the y axis ((response units, RU) over time after start of injection on the axis (s). Association was monitored for 300 s and dissociation for 600 s. **B**. B41 SOSIP trimer was first 2G12-affinity and then SEC-purified and thereafter depleted by passage through Sepharose columns with the bNAbs PGT151 or PGT145 or no antibody on the beads. The flowthrough trimer was immobilized and analyzed by SPR as in A.

The bNAb binding to differentially purified B41 and BG505 SOSIP.664 trimers was then compared by SPR (Fig. 6). Binding to the BG505 SOSIP.664 trimer was indistinguishable after 2G12, PGT145, and PGT151 purification by the bNAbs VRC01, VRC34.01, 35O22 (gp120-gp41 interface [55]), and 3BC315 (gp41, inter-protomeric [56]), as well as by the two purification bNAbs, PGT145 and PGT151, whereas 2G12 and PGT121 binding subtly favored the 2G12-purifed form.

In contrast, the B41 SOSIP.664 trimer showed a wide range of distinct antigenicities resulting from the different purifications (Fig. 6A). VRC01 bound better to the PGT151-purified trimer than to the other two. 2G12 bound marginally better to trimers purified with itself than with the other two bNAbs. Although the PGT121 epitope includes the N332 glycan, which is central to the 2G12 epitope, PGT121 bound most strongly to PGT151-purifed, and least strongly to PGT145-purified trimer; binding to 2G12-purified trimer was intermediate. PGT145 binding to the trimer purified with itself was stronger than to the other two forms. The greatest differences occurred with PGT151 and VRC34.01 binding, the binding to the PGT151-purified trimer strongly dominating, whereas binding to neither of the other two forms was detectable with VRC34.01. Another interface bNAb, 35O22, bound distinctly better to the PGT151-purified form than to the other two, although the difference was smaller than for PGT151 itself and VRC34.01. 3BC315, binding inter-protomerically and closer to the base than the interface bNAbs, gave another distinct ranking: 2G12-purified trimer highest, PGT145-purified somewhat lower, and PGT151-purified markedly lower. Finally, the autologous mNAb 16D bound best to PGT145-purified, more weakly to 2G12-purified, and only to a low level to the PGT151-purified form, indicating, again, that most PGT151-reactive forms in the trimer population expose the 289-glycan-hole epitope less well or present it in less antigenic shape than does the PGT145-purified trimer. The exposure of the 289 epitope may require a flexibility that the most PGT151-reactive forms lack [38].

The affinity-*purified* fractions of trimer correspond to the *eluate* in the chromatography. Because of the distinct antigenic effects of the differential affinity-purification specifically on the B41 trimer SOSIP.664 trimer, we also *depleted* it with bNAb-affinity columns, collecting the *effluent*. Thus, 2G12 and SEC purification of the B41 SOSIP.664 trimer followed by PGT145, PGT151, or mock depletion gave further evidence of antigenic heterogeneity. Depletion with PGT145 reduced PGT145 binding to the trimer and depletion with PGT151 increased it. Conversely and more markedly, depletion with PGT151 reduced PGT151 binding to the trimer and depletion with PGT145 increased it. 2G12 binding was also affected by the depletions: PGT151 depletion enhanced it and PGT145 depletion reduced it (Fig. 6B); these effects suggest allosteric connections between non-overlapping epitopes, as has been described [36, 57]. They are intriguing since no marked corresponding effects were recorded for differential purifications, but the latter may involve not only selection but also persistent induction of conformations.

**Figure 7:**
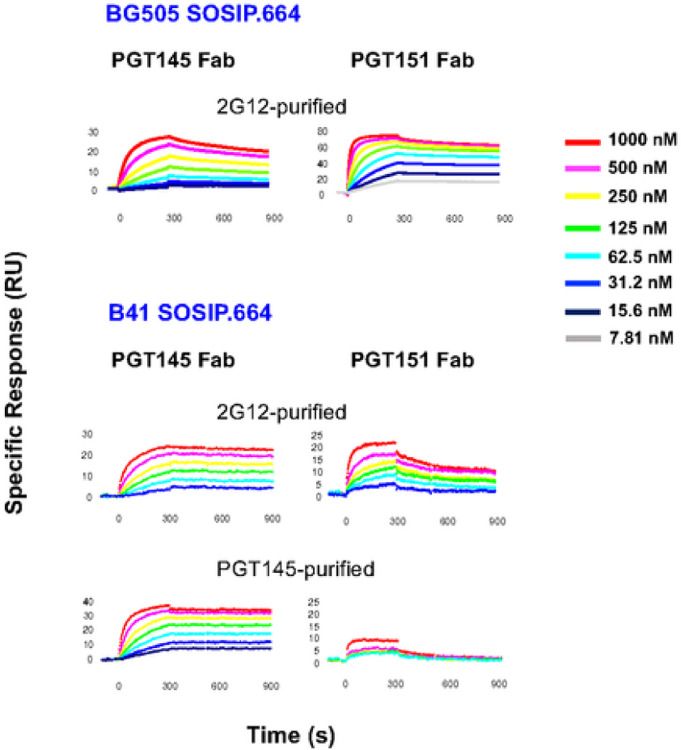
bNAb-Fab binding to BG505 and B41 SOSIP.664 SOSIP,664. Fabs of PTG145 or PGT151 were injected over trimer immobilized freshly in each cycle at concentrations as indicated in the color-coded legend. BG505 SOSIP.664 trimer was 2G12-purifed and B41 SOSIP.664 trimer 2G12- or PGT145-purified, as indicated. Each sensorgram shows the response after background subtraction on the y axis ((response units, RU) over time after start of injection on the axis (s). Association was monitored for 300 s and dissociation for 600 s. The sensorgram for PGT151 Fab binding to PGT145-purified B41 trimer has no model curves.

The sensorgrams for Fab titrations against BG505 and B41 SOSIP.664 trimers are shown in Fig. 7. Kinetic constants *k*_*on*_ and *k*_*off*_, the dissociation constant, *K*_*D*_, and the stoichiometry, *S*_*m*_, were first obtained by Langmuir modeling (Table 1). Langmuir modeling gave passable fits, **χ**^**2**^ ranging from 0.13 to 0.65; the values of the kinetic constants were significant: *T* values (= mean/(s.e.m.)) were > 10, except for *k*_*off*_ of the highly stable PGT145-Fab binding to PGT145-purified B41 SOSIP.664, which fell below the level of detectability, 10^− 5^ (s^− 1^). For the other combinations the *T* values were in the range 51–545 (**SI** Table 1).

The *k*_*on*_ value for PGT151 binding to BG505 SOSIP.664 was higher and the *k*_*off*_ value lower than for PGT145 binding: the net effect was a 14-fold higher intrinsic affinity of PGT151 than PGT145 Fab, in agreement with the higher neutralization potency of PGT151; the partial bivalency of IgG binding to virions is not expected to change IC_50_ ratios but to enhance potency weakly or moderately [3, 30, 58, 59].

The kinetic analysis of binding to B41 SOSIP.664 showed that the PGT145 purification gave a somewhat higher (60%) *k*_*on*,_ a lower *k*_*off*_, falling below detectability, and lower *K*_*D*_, whose upper limit only could therefore be determined, than the corresponding values for 2G12-purified trimer. Those differences suggest an antigenic heterogeneity in the B41 SOSIP.664 population, which is marked after 2G12 purification and diminished by PGT145 purification.

The most central explanatory findings were the differences in stoichiometry between PGT145 and PGT151 binding to BG505 and B41 SOSIP.664 trimers. The stoichiometric *S*_*m*_ value of PGT145 Fab binding to BG505 trimer, 0.74, approached the ideal maximum of 1.0 for this epitope [30, 36, 60]; that of PGT151 Fab binding to BG505 trimer was close to its previously described maximum of 2.0 [30, 38, 61]. PGT145 bound with distinct stoichiometries to 2G12-purified, and PGT145-purified B41 trimer, *S*_*m*_ = 0.63 and 0.87, respectively, showing that the 2G12-purified trimer population contains species lacking detectable affinity for this antibody. Most striking was the low PGT151 stoichiometry for 2G12-purified B41 trimer: 4.2-fold lower than for BG505. PGT151 Fab bound so poorly to PGT145-purified B41 trimer that the data could not be modeled. Taken together, these findings strongly suggest subpopulations among the B41 SOSIP.664-trimer molecules with distinct antigenicities. The heterogeneity manifesting itself as reduced *S*_*m*_ values comprises binding and non-binding forms.

We investigated potential heterogeneities within the binding populations further by applying a heterogeneous-ligand model. Four criteria can be applied to evaluate the meaningfulness of the more complex model: first, a marked reduction in **χ**^**2**^; secondly, *T* values > 10, except if the modeling suggests the existence of a site from which the Fab dissociates below the level of detection; thirdly, that the modeled kinetic parameters for the two sites are distinct; and fourthly, that the component *S*_*m*_ values are not highly distinct, *i.e*., that a minority site is not negligible in population size. The outcome was that meaningful heterogeneity was discernible within the population of BG505- but not the B41-trimer molecules that showed any detectable binding: for BG505, the **χ**^**2**^ values were reduced 2.9- to 6.2-fold; *T* values were high except for a component of extremely slow dissociation of each Fab; *k*_*on1*_*/k*_*on2*_ was 5.8 (PGT145) or 5.0 (PGT151) and *k*_*off1*_*/k*_*off2*_ was > 200 (PGT145) or > 76 (PGT151); *S*_*m1*_*/S*_*m2*_ was 1.0 (PGT145) or 1.7 (PGT151). The complex modeling also increased the stoichiometry for PGT145 from *S*_*m*_ = 0.74 for Langmuir to a cumulative *S*_*m1*_+*S*_*m2*_ = 0.92 for heterogeneous-ligand modeling, a value close to the ideal *S*_*m*_ = 1.0 (Table 2 and **SI** Table 2).

In contrast, only the *T*-value criterion was met for the B41 trimer: the suggested kinetic constants and affinities were close or indistinguishable, and the minority populations small or negligible: specifically, for PGT151 *k*_*on1*_*/k*_*on2*_ was 1.2, *k*_*off1*_*/k*_*off2*_ 1.1, *K*_*D1*_*/K*_*D2*_ 0.82, and *S*_*m1*_*/S*_*m2*_ 110 (**SI** Table 2): the sites did not differ tangibly in kinetics and the minority site was negligible.

In conclusion, two kinds of antigenic heterogeneity were detected: an inclusive kind, prevailing within the binding population of epitopes, was kinetically discernable for BG505; an exclusive kind, dividing the binding from the non-binding population, manifested itself as reduced stoichiometry for B41, very moderately for PGT145 but prominently for PGT151 binding to 2G12-purified trimer.

### Structural modeling of PGT151 binding to the B41 SOSIP.664 trimer

**Figure 8:**
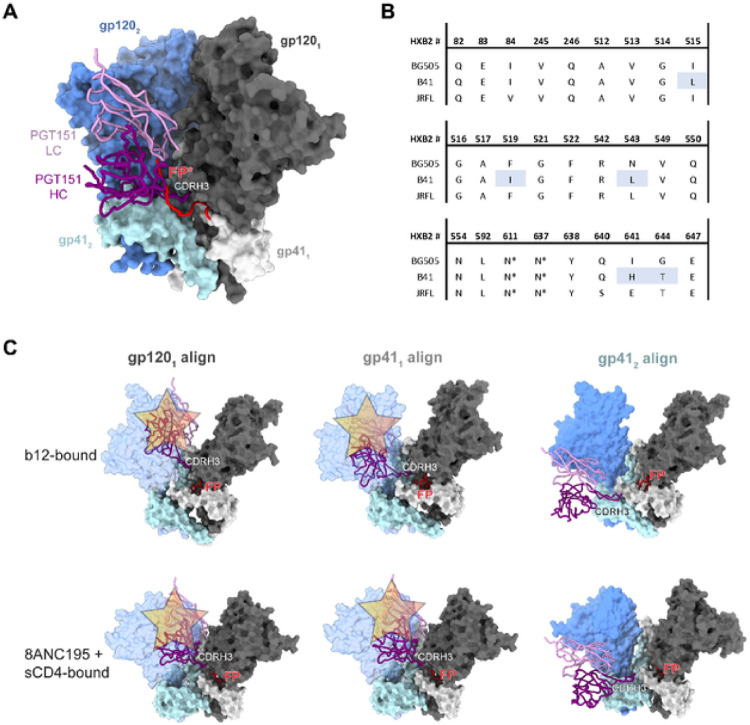
Structural constraints of PGT151 binding to B41 SOSIP.664. **A.** PGT151 (PDB 5FUU; in complex with JRFL Env [72]) aligned to a cryo-EM structure of B41 SOSIP.664 (PDB 6U59 [51]). The fusion peptide is depicted in red and derived from the PGT151+JRFL SOSIP.664 [38] model for reference. **B**. PGT151 contact residues in the epitope (as defined by <4 Å distance from paratope atoms in the PGT151+JRFL SOSIP.664 model) and equivalent positions in BG505 and B41 SOSIP.664 reveal high conservation. Amino acids different between B41 and BG505 are highlighted in light blue. **C**. Alignment of PGT151 Fab from the PGT151+JRFL SOSIP.664 structure onto the b12-bound (top, PDB 5VN8 [26]) or 8ANC195- and sCD4-bound (bottom, PDB 6EDU [28]) conformation using one of three reference chains [26, 28]. Note that for clarity, the b12, 8ANC195, and sCD4 structures have been removed from the reference model. Steric clashes are depicted with translucent buff-colored stars. The clashing gp120 subunit is depicted in transparent blue. For the rest, antibody and Env colors follow those in panel **A**.

Using available structural data, we examined whether the differential PGT151 binding could be caused by pronounced conformational flexibility or “breathing” in B41 SOSIP.664 that would limit access to the PGT151 epitope (Fig. 8). As noted, we previously observed substantial conformational heterogeneity in B41 SOSIP.664 by NS-EM (Fig. 5, [23–26, 52]), as well as the ability of a CD4bs bNAb, b12, to bind to its conformationally dependent epitope on that trimer [26]. Earlier hydrogen-deuterium-exchange mass spectrometry (HDX-MS) experiments demonstrated that although b12 can bind BG505 SOSIP.664 trimers, it does so very slowly, and fails to neutralize the BG505 PV, suggesting that the required more open conformation is rarely sampled by BG505 SOSIP.664 and not triggered by the antibody in a timeframe conducive to neutralization [62]. Hence, the B41 trimer is intrinsically more flexible than the BG505 trimer (Figs. 5 and 8). Double-electron-electron resonance (DEER) spectroscopy has revealed multiple conformations in the trimer base and inner domain of both BG505 and B41 SOSIP trimers, a flexibility that is uncoupled from that of the conformationally more fixed trimer apex [57]. Less conformational heterogeneity in the apex could explain the absence of marked PFs in PGT145 neutralization of either virus (Fig. 1). The same DEER experiments suggested a degree of conformational homogeneity in B41 SOSIP.v4.1, which includes stabilizing mutations to limit exposure of the V3 region and to shield non-NAb epitopes. But we did not study B41 SOSIP.v4.1 or other hyper-stabilized variants here, because we sought to mimic the neutralization-relevant conformational flexibility of Env on virions. We note, however, that the rate of breathing, accordingly, is quite plausibly limited by stabilizing mutations [53, 57]. The conformations of epitopes from apex to base are interconnected in a complex network of long- and short-range effects. For example, pre-binding of PGT145 to the BG505 SOSIP.664 trimer markedly reduces subsequent PGT151 binding, whereas no converse effect is detectable [63]. This non-reciprocal allosteric effect may not be identical for B41, but it suggests an intricate relationship between the two epitopes pertinent to the non-overlapping antigenicity maxima in the trimer population (Figs. 6 and 7; Table 1; **SI** Fig. 1).

The PGT151 epitope is unusually complex: PGT151 binding strictly depends on the native quaternary structure of proteolytically cleaved gp120-gp41 protomers. The paratope closely interacts with the interface between gp120 and gp41 of one protomer and glycans on both subunits of another protomer, inserting itself inter-protomerically [38] (Fig. 8A). Most residues implicated in the interaction are, however, identical for BG505 and B41, including the PNGSs at gp41 positions 611 and 637 (Fig. 8B). Both the fusion peptide (FP) and FP-proximate region (FPPR), reached by the inter-protomeric insertion of the long complementarity-determining region 3 of the heavy chain of the antibody, CDR H3, are highly similar in the two Envs (Fig. 8B). Modeling of PGT151 (based on a complex of its Fab with JR-FL SOSIP.664 [38]) onto a structure of closed-conformation B41 SOSIP.664 does not suggest any clashes or unfavorable interactions that would explain the different stoichiometries (Fig. 8C). Partially open, intermediate conformations of B41 SOSIP.664 have been solved by cryo-EM, including the aforementioned b12-bound state, one in complex with sCD4 and the interface-specific bNAb 8ANC195, and a more rearranged sCD4- and 17b-bound one [26, 28]. Because conformational changes occur both in the gp120 and gp41 subunits in these states relative to the closed conformation, we aligned the PGT151- bound structure in three different ways for three parts of the epitope: to the part of the primary gp120 (gp120_1_), to part of the primary gp41 (gp41_1_), which includes fusion-peptide residues, and to the component of the adjacent gp41 (gp41_2_) at its interface with the paratope (Fig. 8C). In the first two cases, the alignment to the primary subunit component of the epitope results in a clash of PGT151 with the adjacent gp120, which has rotated in response to b12 or sCD4 and 8ANC195 binding (Fig. 8C). This clash alone would prevent binding of PGT151 at this binding angle. Alignment to gp41_2_ relieves the clash but allows paratope contacts with the trimer exclusively by the CDR H3, which is unlikely to yield tangible binding. Lastly, it was shown that, in the b12- or sCD4- and 17b-bound state, the fusion peptide of B41 SOSIP.664 is not solvent-accessible and is instead sequestered in a newly formed pocket in gp41 in all three protomers [26]. Sequestration of the fusion peptide may be a typical response to trimer opening, and this would remove a key component of the PGT151 epitope during such intervals of breathing.

In conclusion, the binding data suggest heterogeneity in how PGT145 and PGT151 recognize the total population of BG505 trimer, but that their binding is sufficient in strength and extent for the antibody to neutralize potently and effectively. Some subtler heterogeneity of PGT145 binding specifically to 2G12-purified B41 trimer was also apparent. Notably, however, the stark difference in stoichiometry of PGT151 binding to the BG505 and B41 trimers explains the larger PF of the B41 than of the BG505 neutralization. The low stoichiometry of PGT151 binding to B41 SOSIP.664 is explained by reduced paratope access through steric hindrance and the sequestration of a key component of the epitope by the partial opening of the trimer, which the B41 SOSIP.664 trimer is prone to.

## Discussion

Studies on incomplete virus neutralization, which leaves distinct PFs, have a long history. Plateaus of maximum neutralization have been observed over time as the NAb association with virions progresses, or as the NAb concentration is increased. Multifarious explanations have been conjectured, but none has held up in general. Genetic resistance of the PF virus has, however, been ruled out in several cases [3, 31–33, 64]. Arguably, both potency and efficacy are important in preventing viral acquisition *in vivo*: a meta-analysis showed that to protect 95% of macaques against SHIV acquisition, the serum reciprocal neutralizing titers needed to be at least ~ 700-fold above the 50%-inhibitory dilution factor, ID_50_ [43]. Provided the Hill coefficient of the sigmoid neutralization curve is equal to 1 [3, 65], those titers would neutralize 99.86% of an inoculum *in vitro*: a PF of 0.14% would translate into 5% failure to protect monkeys from acquisition. But neutralization *in vitro* may differ in many respects from that *in vivo*, and the NAb concentrations in mucosae may be considerably lower than in sera. Still, a PF of several percent, detectable *in vitro*, would arguably augur badly for protection from acquisition *in vivo*. A large PF could thwart protection through preventive passive or active immunization. The importance of the PF in bNAb therapy may be even greater than in prevention, particularly when the aim is eradication: the viral swarm will be considerably more diversified than in the transmission bottleneck [66–68]. And heterogeneity of Env reduces the capacity of inhibitors to block viral entry [41].

We postulate variations in conformation and glycosylation – both occupancy on glycosylation sites and type of glycan - and combinations thereof - as potential sources of antigenic heterogeneity in clonal HIV-1 [26, 57, 69–75]. The conformational heterogeneity could involve both glycans and peptidic segments, directly within the epitope and indirectly through distance effects. The heterogeneity at glycan sites could arise from variable occupancy, type, and processing. Specifically, the heterogeneity would have to be greater in B41 than BG505 to explain the difference in PF in neutralization by PGT151.

Here we describe a PF of B41 neutralization by PGT151 of 21% and a negligible corresponding PF for BG505; PFs for both viruses were minor with PGT145. Incomplete neutralization by PGT151 is not restricted to B41: a range of 60–80% maximum neutralization was measured for 15% of PVs derived from 117 isolates, in contrast to 0.85% by PG9 (our calculations from [39]).

Also noteworthy is the stoichiometry of two PGT151 paratopes per trimer, the ligation of the first two epitopes apparently impeding that of the third [30, 38, 60]. The conformational changes restricting the stoichiometry may also stabilize the trimer [38]. Indeed, the mechanism of neutralization may be a block of conformational changes induced by CD4 and 87 in the absence of the bNAb [3, 4, 38, 76]. And the efficacy of that block may vary differently over trimer populations from different isolates.

The conformational plasticity of B41 Env [25, 26], combined with the allosteric interplay between the PGT145 and PGT151 epitopes, suggests a prominent conformational cause of the B41 PF with PGT151. Our findings of differential antigenicity after different purifications and depletions of B41 trimer, and of differential neutralization of B41 PV after depletion with either bNAb, are highly compatible with a conformational basis of the PF but do not exclude the influence of glycosylation. Glycan heterogeneity does not, however, confer variation in neutralization by all NAbs: it is a hallmark of bNAbs that they can make contacts with the N-acetyl-glucosamine (GlcNAc) stalk of the glycan, while avoiding clashes with high mannose and hybrid glycans of different sizes [36, 77].

We emphasize the possibility of different kinds of heterogeneity: antigenically inclusive and exclusive, *i.e*., modifying kinetics or stoichiometry [78]. Within the population of the SOSIP trimer that does bind PGT145, we detected some heterogeneity by comparing Langmuir and heterogeneous-ligand modeling. The difference between the models was markedly clearer for BG505 than B41 (Tables 1 and 2 and **SI** Tables 1 and 2). Two distinct sites with different kinetics and affinity of binding were discernable for BG505. The combined stoichiometry, however, was high (0.92). If a similar heterogeneity exists on virion-associated Env spikes, these findings could signify that the higher-affinity site gets fully occupied at low concentrations of either bNAb, whereas additional partial occupancy on the lower-affinity site makes the sum sufficient for high efficacy of neutralization.

The glycosylation sites N156 and N160 are both crucial for neutralization by PGT145. N156, however, has indirect effects on the epitope; only the N160 glycan makes direct contact with the paratope [36]. The paratope interacts in an asymmetric manner, extensively with the N160 glycan on a first protomer, less so with the one on a second, and negligibly with the one on a third, the latter glycan projecting away from the paratope [36]. Again, the interaction is driven by the GlcNAc stalk: Man6, Man7, Man8, or hybrid moieties can be tolerated, but probably not bulkier complex glycans. Enough heterogeneity has been described for N160 to explain the observed heterogeneity in binding of the PGT145 Fab to the BG505 trimer: the glycan is partly processed but largely of oligomannose type [36].

The stoichiometry of PGT145 Fab binding was close to the ideal 1.0 for both BG505 and B41. PGT151 binding also approached its described maximum stoichiometry of 2.0 on the BG505 trimer. In contrast, on the B41 trimer the PGT151 stoichiometry was a mere 0.45 after 2G12 purification, and the binding was barely detectable after PGT145 purification (Table 1). A complete lack of binding to a large fraction of the trimer molecules explains the large PF for the B41-PGT151 combination. But the proportion of sensitive virus cannot be directly derived from the stoichiometry: that proportion will be determined by the distribution of the antigenic forms of trimers on the virions combined with approximate thresholds of minimum occupancy for neutralization [79].

The binding of a bNAb may alter the conformational heterogeneity among the trimer molecules. But a large fraction of primarily non-antigenic Env epitopes that could be induced to fit the paratope would conceivably not confer a large PF and would leave tell-tale marks on the SPR-curve shapes [30]. Less malleable antigenic heterogeneity is required to explain the PF.

The armamentarium of passive immunization offers straightforward remedies for large PFs caused by antigenic heterogeneity: combinations of bNAbs that have distinct preferences for glyco-forms or conformational variants of the antigen [46, 80, 81]. Do the results also inform active immunization? In cases of reciprocal enrichment and depletion of antigenic forms by affinity purification with certain antibodies, such as PGT145 and PGT151, the immune responses might be skewed towards resembling the antibody used for purification of the immunogen. But elicitation of responses to apical and interface bNAb epitopes are still not readily achieved by immunogens purified with these bNAbs. The goal of eliciting responses to multiple bNAb epitopes, however, might still be favored by immunogens purified with bNAbs such as 2G12 that largely do not segregate distinct antigenic forms of the immunogen (before SEC) or by combinations of differentially purified immunogen.

To elicit bNAbs, it may be crucial to avoid other, potentially distracting responses, such as narrowly active autologous NAbs. B41-autologous NAbs tend to target the lining of holes in the glycan shield around residues N230 and N289 [20,51]. Such epitopes, targeted by the rabbit mono- and polyclonal antibodies we studied here, were less exposed on the PGT151- than the PGT145-purified trimer. These rabbit antibodies neutralized PGT151-depleted PV more potently and effectively than PGT145-depleted PV. PGT151 purification also reduced PGT145 binding specifically to the B41 SOSIP.664 trimer much less than *vice versa*. Those skewed specificities would suggest advantages to PGT151- over PGT145-purified immunogen. But inducing bNAbs that leave as large a PF as PGT151 does against many isolates would not optimal [39]. Therefore again, combinations of antigenic variants may be more conducive to rendering responses broader and more effective.

We conclude that antigenic heterogeneity within genetically homogeneous Env-protein populations can differ drastically among HIV-1 strains, which is directly exemplified here and in line with data on multiple isolates [39]. The ensuing effects on neutralization efficacy and potency can be strong. Countermeasures can be designed both in passive and active immunization. In the latter case, the heterogeneity may even be harnessed in the pursuit of breadth.

## Methods

### Aim, design, and setting

The aim of this study was to find explanations for reduced efficacy in HIV-1 neutralization. We compared the neutralization of the Clade A BG505 isolate with that of Clade B B41 by three bNAbs, 2G12, PGT145, and PGT151, as well as post-immunization rabbit poly- and monoclonal antibodies. We observed large PFs specifically with B41 and PGT151 and the rabbit antibodies. We dissected the heterogeneity by performing neutralization of bNAb- or mock-depleted PV and studies of bNAb binding to bNAb-purified SOSIP trimers by ELISA and SPR. We correlated the findings with available protein-structural and glycosylation data.

### Antibodies

The human bNAbs VRC01, 2G12, PGT121, and PGT145 [35–37, 82] were obtained from the International AIDS Vaccine Initiative (IAVI, La Jolla), VRC34.01 [48] from John Mascola (VRC, NIH), 35O22 [83] from Mark Connors (VRC, NIH), and the rabbit mNAbs 16D and 13A from Marit J van Gils (AMC). The bNAb 3BC315 was produced by us, as described [56].

PGT145- and PGT151-Fab expression plasmids were expressed and purified as described previously [60]. Briefly, HEK 293F suspension cells were transiently transfected with Fab plasmids. Fabs were initially purified on anti-human affinity column (kappa XL matrix). Fabs were further subjected to ion-exchange fractionation by AKTA FPLC to remove dimers of light chains. The purity of the Fabs was confirmed on SDS PAGE gel (reducing and non-reducing) before binding analyses.

### Pseudo-virus production

HEK-293T cells were transfected with HIV-1 BG505 and B41 *env* and luciferase-reporter plasmids [24, 84] to produce pseudo-virus (PV). B41 Env for the PV has an R315Q mutation in the V3 region to make it similar to B41 SOSIP.664, which has the substitution to prevent proteolytic clipping [25]. One day before transfection, cells maintained in growth medium (DMEM with 10% FBS, 2mM L-Glutamine and 1% Pen-Strep) were seeded in 6-well plates at a density of 4 · 10^5^ cells per well. From 3h before transfection 50–60%-confluent cell cultures were maintained in antibiotic-free growth medium. The HIV-1 BG505 or B41 env in pCDNA3 and pNL4.1AMΔ*env*Δ*vpr* + *luc* plasmids [85] at a ratio of 1:2 were mixed with the Effectene Transfection Reagent (Qiagen) and the mix was added to the cultures, which were incubated at 37°C with 5% CO_2_. The next day, cells were supplemented with fresh growth medium. 48h thereafter, PV-containing supernatants were harvested and spun at 2000rpm for 10 minutes. Additional FBS to a total of 20% was added to the supernatant, before spinning, to avoid virion degradation.

### TZM-bl neutralization assay

The persistent fraction of infectivity after neutralization of BG505 and B41 PV was measured, and the neutralization of PGT145-, PGT151-, and mock-depleted B41 PV (see below) was compared in a neutralization assay based on TZM-bl cells [86]. Initially in this study, neutralization assays were performed as described previously [24]. Subsequently, after a lab-routine switch during the pandemic, we used a slightly different TZM-bl-PV system [85], yielding indistinguishable results. Briefly, the day before infection, cells were seeded in 96-well plates, at a density of 1 · 10^4^ cells per well. PV at a dose yielding luminescence readouts of ~ 2 · 10^6^ counts per second was incubated for 1h at 37°C with 5% CO_2_ with select bNAbs or heat-inactivated serum samples, all serially diluted in steps in DMEM growth medium. 50μl mix or medium only (cell control) was then transferred to TZM-bl cells seeded the night before (4 replicates) in wells with 50μl medium or to wells with 50μl medium without cells (viral-input control, 2 replicates), all wells containing 15μg/ml DEAE-Dextran. Two days later, the medium was carefully aspirated and the cells were lysed with Glo-lysis buffer (Promega E153A) during 15 minutes on a shaker. 25 μl Nano-Glo Dual-Luciferase substrate (Promega 1610) was added to each well. Luciferase-generated signal was read with an Enspire multimode plate reader (Perkin Elmer). Data were processed by subtracting the background signals of cell- and virus-only from sample wells. Virus incubated without antibody was considered to give 100% infectivity and percent inhibition was calculated for all samples.

In a variation of the standard set-up, serially diluted PV was instead incubated with a constant concentration of antibody (50μg/ml) or with medium for 1 hour at 37°. The PV-bNAb mix was added to the Tzm-bl cells, the cultures continued; on day 2 post-infection, cells were washed and lysed, and signals generated and measured as above. Neutralization data were plotted and analyzed with GraphPad Prism 6 software.

### Depletion of PV with bNAbs

Supernatants with PV were added to columns containing Sepharose 4B beads with CNBr-coupled PGT145, PGT151, or no antibody (as mock control). PV mixed with beads were incubated at 37°C with 5% CO_2_ for 3h on a nutator. After 3h, beads were allowed to settle down at room temperature. PV was collected by gravity flow from the respective column, filtered through 0.45μm membranes, concentrated by Vivaspin columns with a 100-kDa cut off (Cytiva), and immediately tested in the neutralization assay.

### Expression, purification, and fractionation of SOSIP trimers

BG505 and B41 SOSIP.664-His genes were cloned into the pPPI4 expression vector (GenScript). Expi-293F cells were transiently transfected by FectoPRO (VWR). Cells were seeded at a density of 4 · 10^6^ cells/ml in 250ml of medium with 1 · penicillin-streptomycin (Corning) one day before transfection. On the day of transfection, cells with 90% viability were suspended in antibiotic-free medium at 6 · 10^6^ cells/ml. For transfection, HIV-1 *env* and *furin* plasmids were diluted in Optimum at a ratio of 4:1, mixed with FectoPRO reagent, and incubated at room temperature for 10 minutes. The FectoPRO-DNA mix and FectoPRO Booster were added to the cells. Transfected cells were incubated at 37°C with 5% CO_2_ and continuous shaking for 3 days. After 3 days, cells were centrifuged at 3000 rpm for 30 minutes. Supernatant was collected and passed through a 0.2-μm filter.

Filtered supernatant was first passed through a 2G12-, PGT145-, or PGT151-affinity column. Affinity columns were made by cross-linking bNAbs to activated CNBr Sepharose 4B beads (GE Healthcare). Bound trimer was eluted with 3M MgCl_2_, which was then removed by dialyzing through snake-skin tubing in an exchange buffer (TN150: 20mM Tris-HCl, 150mM NaCl, pH 8) overnight at 4°C. Further purification of the 2G12-purified trimer was done by size-exclusion chromatography (SEC) on a HiLoad 16/600 Superdex 250 preparative-grade column with TN150 as running buffer to remove aggregates, dimers, and monomers. TN150 was used as running buffer during SEC. Protein concentrations were determined by the bicinchoninic assay (BCA). Trimer content in SEC fractions was assessed by Blue Native-PAGE; pure trimer fractions were pooled and stored at −80°C till further analysis by NS-EM.

2G12- and PGT145-affinity purifications followed by SEC have been shown multiple times by negative-stain electron microscopy (NS-EM) to give BG505 and B41 SOSIP.664 trimers with nearly exclusively native-like structure [17, 22–25, 30, 53]. Here we confirmed by the same method, performed as previously described [24], that PGT151 purification of B41 SOSIP.664 trimer also gave 100% native-like trimer (see Results and Fig. 5).

The BG505 and B41 SOSIP.664 trimers were also affinity-purified for antigenic analyses (Fig. 6, 7, **SI** Fig. 1, Tables 1 and 2). Briefly, supernatant from the 293-transfection cultures was collected, passed through a 0.2-μm filter, and then through 2G12-, PGT145-, and PGT151-affinity columns. Bound trimer was eluted with 3M MgCl_2_, which was removed as above (55). SEC was then performed and trimer fractions identified and stored as above.

In addition, a portion of the 2G12-SEC-purified B41 SOSIP.664 trimer was PGT145- and PGT151-depleted. The trimer was resuspended in TN150 buffer and incubated in PGT145-, PGT151-, or mock-affinity columns at room temperature for 2 hours, with constant nutating. After 2 hours, the unbound, depleted fraction of trimer was allowed to flow through the column. Effluent was collected and concentrated on Viva Spin columns. The quality and purity of all fractions were checked by BN-PAGE and the protein concentration determined by the bicinchoninic-acid assay.

### Analysis of antibody binding by ELISA

Lectin-capture ELISA for analyzing antibody binding to Env SOSIP trimers has been previously described [61]. It was chosen here because of poor immobilization of the His-tagged trimer to Ni^2+^ plates. 96-well plates were coated with GNL-lectin (Sigma) at 5μg/ml in 100μl per well by overnight incubation. All steps were performed at room temperature. The next day, wells were washed once with Tris-buffered saline (TBS) and blocked for 1 hour with 10% FBS and 1% skimmed milk in 200μl TBS. The plates were then washed twice with TBS and B41 SOSIP.664 trimer at 2μg/ml was captured by the lectin for 2 hours. The plates were washed twice and antibody serially diluted in TBS with 5% FBS and 1% skimmed milk was added. After 1 hour, the plates were washed thrice with TBS. Then goat anti-human-HRP or anti-rabbit-HRP conjugate was added. After 45 minutes, the plates were washed four times with TBS containing 0.05% tween-20. Then 3,3′,5,5′-tetramethylbenzidine (TMB) was added for 2–5 minutes and finally 0.3M HCl to stop the reaction. The OD was recorded at 450nm.

### Analysis of antibody binding by surface plasmon resonance

Antibody binding to purified and fractionated SOSIP.664 trimers was also analyzed by SPR on BIAcore 3000 and T200 instruments at 25°C [30]. Briefly, trimers were immobilized to *R*_*L*_ values close to 250 response units (RU) by anti-His antibody that had been covalently coupled to a CM5 sensor chip as described [60, 87]. In each cycle, fresh Env protein was captured, and at the end of each cycle, Env trimer was removed by a pulse of 10mM glycine (pH 2.0) for one minute at a flow rate of 30 μl min^− 1^. IgG of bNAbs at a concentration of 500nM was injected for 300s of association and 600s of dissociation. For full kinetic analysis, Fabs of PGT145 and PGT151 were titrated down from 1μM in 2-fold steps till absence of detectable signal. Significant mass-transfer limitation was prevented by a high flow rate (50 μl/min); its absence was confirmed by *k*_*t*_ analysis. Binding data were analyzed with BIAevaluation software. Data from Fab titrations were fitted to Langmuir and heterogeneous-ligand models. The binding of PGT151 Fab to 2G12-purified B41 SOSIP was modeled with baseline drift (2–7. 10^− 3^ RU/s) because of slight trimer dissociation from anti-His-capture antibody. The modeling was validated by calculations of *χ*^2^ for the overall fit and *T* values for the individual fitted parameters (**SI** Tables 1 and 2).

## Figures and Tables

**Table 1:** Langmuir modeling^a^

SOSIP.664 trimer	Fab	Affinity purification	*k*_*on*_^b^ (1/Ms)	*k*_*off*_^b^ (1/s)	*K*_D_^b^ (nM)	*S*_*m*_^b^
**BG505**	PGT145	2G12 (n = 3)	1.8. 10^4^ ± 3.7. 10^2^	5.1. 10^−4^ ± 2.0. 10^−6^	29 ± 0.69	0.74 ± 6.8. 10^−3^
	PGT151	2G12 (n = 2)	8.7. 10^4^ ± 3.5. 10^2^	1.8. 10^−4^ ± 5.0. 10^−7^	2.0 ± 1.3. 10^−2^	1.9 ± 7.5. 10^−3^
**B41**	PGT145	2G12 (n = 3)	2.0. 10^4^ ± 8.8. 10^2^	1.2. 10^−4^ ± 1.5. 10^−5^	6.2 ± 0.28	0.63 ± 1.6. 10^−2^
		PGT145 (n = 3)	3.2. 10^4^ ± 2.3. 10^3^	< 10^−5^	<0.5	0.87 ± 4.8. 10^−2^
	PGT151	2G12 (n = 3)	4.0. 10^4^ ± 4.1. 10^3^	3.1. 10^−3^ ± 2.4. 10^−4^	77 ± 5.0	0.45 ± 1.5. 10^−2^
		PGT145 (n = 2)	Minimal binding: < 10 RU			

a Tabulated values are means ± S.E.M of *n* independent replicates

b *k*_*on*_ and *k*_*off*_ are the on- and off- rate constants, respectively; *K*_*D*_ is equilibrium dissociation constant = *k*_*off*_/*k*_*on*_. *Sm* is stoichiometric number. *S*_*m*_ denotes the stoichiometry, *i.e*., number of Fab molecules per trimer.

**Table 2:** Heterogeneous-ligand modeling^a^

SOSIP.664 trimerr	Fab	Affinity purification	*k*_*on1*_^b^	*k*_*off1*_^b^	*k*_*on2*_^b^	*k*_*off2*_^b^	*k*_*D1*_^b^	*k*_*D2*_^b^	*S*_*m1*_^b^	*S*_*m2*_^b^	*S*_*m1*__+_*S*_*m2*_^b^
BG505	PGT145	2G12 (n = 3)	2.9. 10^4^ ± 7.5. 10^2^	< 10^−5^	5.0. 10^3^ ± 7.0. 10^2^	2.0. 10^−3^ ± 9.1. 10^−5^	<0.5	4.1. 10^2^ ± 64	0.46 ± 1.6. 10^−2^	0.46 ± 2.4. 10^−2^	0.92 ± 3.8. 10^−2^
	PGT151	2G12 (n = 2)	1.4. 10^5^ ± 1.3. 10^3^	< 10^−5^	2.8. 10^4^ ± 1.0. 10^3^	7.6. 10^−4^ ± 2.5. 10^−5^	<0.1	27 ± 1.9	1.2 ± 1.9. 10^−2^	0.70 ± 1.1. 10^−2^	1.9 ± 7.7. 10^−3^
B41	PGT145	2G12 (n = 3)	5.2. 10^4^ ± 1.4. 10^4^	1.9. 10^−4^ ± 3.6. 10^−5^	1.6. 10^4^ ± 1.5. 10^3^	1.1. 10^−4^ ± 1.7. 10^−5^	3.7 ± 0.23	6.6 ± 0.80	4.4. 10^−2^ ± 4.4. 10^−2^	0.59 ± 3.6. 10^−2^	0.63 ± 1.8. 10^−2^
		PGT145 (n = 3)	3.5. 10^4^ ± 1.4. 10^4^	< 10^−5^	4.5. 10^4^ ± 1.1. 10^4^	< 10^−5^	<0.5	<0.5	0.13 ± 0.13	0.74 ± 0.16	0.87 ± 5.1. 10^−2^
	PGT151	2G12 (n = 2)	3.1. 10^4^ ± 1.9. 10^3^	8.5. 10^−4^ ± 2.5. 10^−4^	2.5. 10^4^ ± 5.5. 10^3^	7.8. 10−4 ± 9.8. 10−5	28 ± 9.8	34 ± 12	0.45 ± 2.9. 10^−2^	4.1. 10^−3^ ± 4.0. 10^−3^	0.45 ± 2.5. 10^−2^

a Tabulated values are means ± S.E.M of *n* independent replicates

b The constants and *S*_*m*_ are explained in Table 1 but here heterogeneous model allocates distinct values for two different sites, each indicated by the subscript 1 or 2. *S*_*m1* +_*S*_*m2*_= the cumulative stoichiometry.

## Data Availability

all relevant data are contained in the article including its supplementary information; Protein Databank Coordinates for previously published structural data that were used are provided in the legend to Figure 8 (PDB 5FUU, PDB 6U59, PDB 5VN8, and PDB 6EDU).
